# Nomophobia, Insomnia, and Coping Strategies in Relation to Academic Achievement and Psychological Well-Being Among Medical Students: A Cross-Sectional Study

**DOI:** 10.3390/bs16081379

**Published:** 2026-08-11

**Authors:** Lucia Terze, Anni Klapez, Ivana Petricevic, Ivana Pavlinac Dodig, Renata Pecotic, Zoran Dogas

**Affiliations:** 1 Health Center of Split-Dalmatia County, Kavanjinova 2, 21000 Split, Croatia; lucia.terze@dz-sdz.hr (L.T.); anni.klapez@dz-sdz.hr (A.K.); ipetricevic1998@gmail.com (I.P.); 2 Department of Neuroscience and Sleep Medicine Center, University of Split School of Medicine, Soltanska 2A, 21000 Split, Croatia; renata.pecotic@mefst.hr (R.P.); zoran.dogas@mefst.hr (Z.D.)

**Keywords:** medical students, nomophobia, insomnia, psychological well-being, coping strategies, academic performance, smartphone addiction, student mental health

## Abstract

Medical students are exposed to substantial academic and psychological demands that may influence their well-being, lifestyle behaviors, and academic performance. This cross-sectional study aimed to examine the associations between smartphone-related anxiety (nomophobia), insomnia symptoms, psychological well-being, coping strategies, and academic achievement among medical students. The study was conducted among 216 students (mean age 22.03 ± 2.48 years, 72.2% female) at the University of Split School of Medicine. Data were collected through an anonymous digital questionnaire created in Google Forms, assessing general information and including the International Physical Activity Questionnaire, Nomophobia questionnaire, Insomnia Severity Index, Medical Student Well-Being Index, and Brief COPE questionnaire. Academic performance was assessed using students’ self-reported arithmetic and weighted grade point averages (GPAs), which were analyzed separately. There was a high prevalence of nomophobia and reduced well-being among participants. Nomophobia was positively associated with insomnia severity (r = 0.172, *p* = 0.011) and poorer psychological well-being (r = 0.268, *p* < 0.001), while insomnia severity (r = −0.172, *p* = 0.011) and avoidant coping strategies (r = −0.167, *p* = 0.014) were negatively correlated with academic performance. The examined demographic, lifestyle, digital, and psychological variables explained a modest 9.0% of the variance in students’ weighted GPAs, which is consistent with the multifactorial nature of academic achievement. These findings suggest that digital dependence, insomnia symptoms, coping styles, and psychological well-being are interrelated and may be relevant to academic functioning among medical students.

## 1. Introduction

A healthy lifestyle encompasses a set of health-related behaviors shaped by both individual choices and environmental opportunities (Cockerham et al., 2020). Previous research has shown that physical activity, reduced sedentary behavior, and limited screen time positively influence mental health, thereby indirectly improving academic performance (Maitiniyazi et al., 2025). Physical exercise significantly contributes to maintaining physical and mental health through enhancing brain function, resulting in improved learning and memory (Yang, 2019; Chang et al., 2012). In the long term, aerobic exercise increases neuroplasticity and cognitive functions (Yang, 2019; Chang et al., 2012; Hötting & Röder, 2013), while acute or anaerobic activity provides short-term benefits, including improved mood, attention, and better cognitive performance (Clemente-Suárez et al., 2023). Students with high physical fitness are less likely to have poor academic performance than those with low physical fitness, while a sedentary lifestyle may adversely affect academic functioning (Redondo-Flórez et al., 2022; Burrows et al., 2017). However, the relationship between prolonged sedentary behavior and academic performance may depend on the context in which sedentary time occurs. Moreover, prolonged sedentary behavior has emerged as an independent lifestyle factor associated with adverse physical and mental health outcomes (Bull et al., 2020; Firth et al., 2020). Medical students spend a substantial proportion of their day sitting during lectures, studying, and screen-based learning activities. As a result, excessive sedentary time has been associated with poorer mental health, reduced cognitive performance, and impaired academic functioning, although its relationship with academic achievement remains complex and may depend on whether sitting time reflects academically engaged or passive sedentary behaviors (Pengpid & Peltzer, 2019; Richardson et al., 2012; Redondo-Flórez et al., 2022). Therefore, both physical activity and sitting time were included in the present study as complementary indicators of students’ lifestyle behaviors.

The widespread popularity and excessive use of smartphones can lead to problematic addictive behaviors, which disrupt general well-being and have negative effects on mental health (Paterna et al., 2024). Problematic smartphone use has increasingly been conceptualized as a form of maladaptive digital behavior characterized by difficulties in self-regulation, excessive reliance on digital devices, and compulsive use despite negative consequences (Paterna et al., 2024; Yildirim & Correia, 2015; Al-Mamun et al., 2025). Within this framework, nomophobia represents a specific manifestation of problematic smartphone use. This psychological condition, common among university students, is characterized by anxiety arising from the inability to access or use one’s mobile phone (Bhattacharya et al., 2019). Noted negative impacts of nomophobia on the social, psychological, and health domains include increased stress levels, lower self-esteem, depression symptoms, reduced quality of life, and challenges with day-to-day functioning (Al-Mamun et al., 2025).

Healthy sleep is essential for overall health and quality of life, as well as for normal neurocognitive functioning and emotional regulation (Oliveira et al., 2025). In academic settings, a demanding environment and irregular schedules often lead to impaired sleep and frequent sleep disturbances (Dąbrowska-Galas et al., 2021). High stress levels, late bedtime, and sleep deprivation are associated with a higher prevalence of sleep disorders among medical students (Oliveira et al., 2025; Dąbrowska-Galas et al., 2021). Furthermore, sleep quality, duration, and consistency are correlated with grade point averages (GPAs) among college students (Okano et al., 2019), while sleep deprivation is associated with impaired cognitive functioning (Hartmann & Prichard, 2018). Given their high prevalence and potential consequences for students’ health and functioning (Dąbrowska-Galas et al., 2021), insomnia symptoms represent an important factor to consider when examining academic performance and psychological well-being among medical students.

Medical students experience disproportionately high levels of stress, depression, and psychological distress, driven by heavy workload, constant evaluations, intense competition, and the overwhelming volume of required information (Rotenstein et al., 2016). Prolonged exposure to such stressors may lead to emotional strain, reduced well-being, and burnout, which can negatively affect motivation, daily functioning, and academic performance (Ishak et al., 2013). Reduced well-being among medical students has been linked to emotional exhaustion, decreased academic engagement, and impaired professional development (Rotenstein et al., 2016). Coping strategies, conceptualized as cognitive and behavioral efforts aimed at managing internal or external demands exceeding one’s resources (Lazarus & Folkman, 1984), may be adaptive or associated with higher psychological distress and less favorable outcomes (Eisenberg et al., 2012). Greater use of adaptive coping strategies was associated with higher academic performance, suggesting that deliberate regulation of emotions and impulses in response to academic stress may confer academic benefits by supporting sustained engagement, effective goal pursuit, and adaptive responses to academic demands (Kassaw & Demareva, 2025).

Although each of these factors has been independently associated with academic performance, limited research has examined their combined contribution within a single model among medical students. Thus, this study aimed to examine the associations among nomophobia, insomnia symptoms, psychological well-being, coping strategies, physical activity, and academic performance in medical students. We hypothesized that higher nomophobia, more severe insomnia symptoms, greater psychological distress, and greater reliance on maladaptive coping strategies would be associated with poorer academic performance. In addition, we explored whether these variables would independently contribute to academic performance after adjustment for demographic and lifestyle factors using hierarchical regression analysis.

## 2. Materials and Methods

This cross-sectional study was approved by the Ethics Committee of the University of Split School of Medicine (Class: 029-01/24-02/0001; Reg. No.: 2181-198-03-04-24-0032). The study included 216 students from all six years of the University of Split School of Medicine.

Data were collected through an anonymous digital questionnaire created in Google Forms. Because the survey was conducted anonymously and no personal identifiers were collected, individual participants could not be re-identified. Data were stored in a password-protected electronic database accessible only to the research team and were used exclusively for research purposes. All data were processed in accordance with applicable data protection regulations. The inclusion criterion was enrolment as a medical student at the University of Split School of Medicine at the time of the study. No specific exclusion criteria were applied. Because all questionnaire items in Google Forms were mandatory, questionnaires with missing responses could not be submitted. Prior to analysis, all submitted questionnaires were reviewed for completeness and consistency. No technical restrictions limiting responses to one Google account or IP address were implemented, and no minimum completion time threshold was applied to exclude potentially careless responses. To minimize social desirability bias, the questionnaire was completed anonymously. Participants were informed that no personally identifiable information would be collected and that all responses would remain confidential. Participants completed the questionnaire independently without investigator influence. Participants provided informed consent after reading the study information and confirming their participation by selecting the appropriate option at the beginning of the questionnaire. The data collection period lasted from 1 March to 21 June 2024. The survey was initially distributed online, but due to a low response rate, it became necessary to approach students in class and invite them directly to participate in the study. The same anonymous questionnaire was used for both recruitment strategies. Of the 540 eligible medical students, 100 completed the questionnaire following the initial online invitation. Subsequently, 300 students were approached during scheduled classes, resulting in an additional 116 completed questionnaires (response rate 38.7%). Due to attendance patterns and scheduling constraints, not all eligible students could be approached during scheduled classes. Overall, 216 students participated in the study, corresponding to an overall response rate of 40.0%. No a priori sample size calculation was performed, as the study aimed to recruit all eligible medical students available during the data collection period. A sensitivity power analysis and a post hoc power analysis were subsequently conducted for the final multiple regression model. The sensitivity analysis for the final multiple regression model (12 predictors, N = 216, α = 0.05) indicated that the study had 80% power to detect an overall effect size of at least f^2^ = 0.084. Additionally, the achieved power to detect a medium effect size (f^2^ = 0.15) was 98%.

The first part of the questionnaire collected general information about the participants, including demographic, anthropometric, and academic characteristics (age, gender, race, year and duration of study, GPAs, body height and weight, and chronic disease information). Academic performance was assessed using students’ self-reported arithmetic and weighted grade point averages (wGPA). Because the survey was conducted anonymously, it was not possible to verify these data against official university records while preserving participants’ confidentiality.

The second part of the questionnaire focused on physical activity. For this section, the long version of the International Physical Activity Questionnaire (IPAQ) was used (Craig et al., 2003). IPAQ evaluates physical activity across five domains: work, transportation, household activities, recreation, and time spent sitting. Scoring for IPAQ is based on calculating MET-minutes (metabolic equivalents), using standardized values: walking = 3.3 METs, moderate activity = 4.0 METs, vigorous activity = 8.0 METs. Total MET-minutes were calculated by multiplying the duration of each activity by its corresponding MET value and summing the results. Based on total weekly MET-minutes, participants were categorized into three levels of physical activity: low (less than 600 MET-min/week), moderate (600–2999 MET-min/week), and high (3000 or more MET-min/week).

In the third part, the Nomophobia questionnaire (NMP-Q) was used to gather information on smartphone use habits (Yildirim & Correia, 2015). Participants responded to 20 items using a 7-point Likert scale, where 1 represents “strongly disagree”, and 7 represents “strongly agree”. After completing the questionnaire, the results were summed and ranged from 20 to 140. A total score of 20 or less indicates the participant does not have nomophobia; scores of 21 to 59 indicate mild nomophobia; 60 to 99, moderate nomophobia; and 100 to 140, severe nomophobia.

Participants were also screened for insomnia using the Insomnia Severity Index (ISI), in which respondents rated the nature and symptoms of their insomnia problems using a Likert scale (Morin et al., 2011). The questionnaire is structured into three parts: the first assesses the severity of insomnia-related problems, the second evaluates satisfaction with sleep patterns, and the third examines the extent to which insomnia affects daily functioning and how noticeable it is to others. The responses are scored on a scale from 0 to 4, and a higher score indicates more severe insomnia symptoms.

Student well-being was assessed using the Medical Student Well-Being Index (MSWBI), a validated screening instrument comprising 7 dichotomous (yes/no) items assessing burnout, emotional distress, depressive symptoms, fatigue, and reduced functioning (Dyrbye et al., 2010). Each affirmative response was scored as 1 point, and each negative response as 0 points, yielding a total score ranging from 0 to 7. Higher MSWBI scores indicate poorer well-being and greater distress, whereas lower scores indicate better well-being and lower distress, with the cut-off value of 4.

Coping strategies were assessed using the Brief COPE questionnaire, consisting of 28 items designed to measure effective and ineffective coping responses to stress (Carver, 1997). The questionnaire is structured into three higher-order coping styles: problem-focused coping (items 2, 7, 10, 12, 14, 17, 23, 25), emotion-focused coping (items 5, 9, 13, 15, 18, 20, 21, 22, 24, 26, 27, 28) and avoidant coping (items 1, 3, 4, 6, 8, 11, 16, 19). Responses are rated on a 4-point Likert scale ranging from 1 (“I usually don’t do this at all”) to 4 (“I usually do this a lot”). Scores for each coping style were calculated as the mean value (sum of item scores divided by the number of items within the coping style). Mean scores ranged from 1 to 4, with higher scores indicating more frequent use of the respective coping style and lower scores indicating less frequent use. No standardized cut-off values were applied to classify participants according to a predominant coping style; instead, all three coping dimensions were treated as continuous variables in the statistical analyses.

Data collected via Google Forms was exported to Microsoft Excel (Microsoft Office 365) for data cleaning and preparation. Statistical analyses were conducted using the MedCalc (version 19.1.2) and Jamovi (version 2.6). Sensitivity and post hoc power analysis were conducted using G*Power (version 3.1.9.7). Descriptive statistics (means, standard deviations, medians, and frequencies) were used to summarize the data. Differences between the groups were analyzed using Student’s *t*-test, associations between continuous variables were assessed using Pearson’s correlation coefficient, and relationships between categorical variables were examined using the chi-square (χ^2^) test. Multicollinearity was assessed using variance inflation factors (VIFs) and tolerance values. A hierarchical multiple regression analysis was conducted to examine the independent associations of demographic, lifestyle, digital, and psychological variables with students’ cumulative wGPA. Regression coefficients are presented with their corresponding 95% confidence intervals. For the hierarchical regression analysis, gender was coded as 1 = male and 2 = female and entered as a binary categorical variable. The three participants who reported another gender identity were excluded because their small number did not allow reliable estimation of regression coefficients. Statistical significance was set at *p* < 0.05.

## 3. Results

### 3.1. Participant Characteristics

A total of 216 medical students from all six academic years participated in the study. The sample included 57 (26.4%) male and 156 (72.2%) female students, while 3 (1.4%) participants reported another gender identity. The mean age of participants was 22.03 ± 2.48 years. Mean arithmetic GPA was 4.16 ± 0.43, while wGPA was 3.85 ± 0.55. The mean duration of study was 4.26 ± 2.15 years (Table 1).

### 3.2. Psychological and Behavioral Measures

The mean nomophobia total score was 71.35 ± 25.43. A total of 2 (0.9%) participants had no nomophobia (nomophobia total score was <21), 67 (31.0%) had mild nomophobia (total score was 21–59), 119 (55.1%) had moderate nomophobia (total score 60–99), and 28 (13.0%) had severe nomophobia (total score 100–140). The mean ISI score was 7.32 ± 5.18. A total of 122 participants (56.5%) had no insomnia, 74 (34.3%) had subthreshold insomnia, 18 (8.3%) had moderate, and 2 (0.9%) had severe clinical insomnia. The MSWBI score averaged 3.27 ± 1.89, and 110 (50.9%) students scored ≥ 4, indicating poor well-being. Regarding coping strategies, participants reported greater use of problem-focused coping (1.93 ± 0.56) than emotion-focused coping (1.68 ± 0.47) and avoidance strategies (0.77 ± 0.46). The psychological and behavioral measures are presented in Table 2.

Internal consistency was good to excellent across all study instruments. For the NMP-Q, both Cronbach’s α and McDonald’s Ω were 0.953. For the ISI, Cronbach’s α was 0.859, and McDonald’s Ω was 0.864. For the MSWBI, Cronbach’s α was 0.714, and McDonald’s Ω was 0.705. For the Brief COPE, Cronbach’s α and McDonald’s Ω were 0.796 and 0.801 for problem-focused coping, 0.703 and 0.720 for emotion-focused coping, and 0.733 and 0.756 for avoidant coping, respectively.

### 3.3. Correlation Analysis

The correlation analysis presented in Table 3 demonstrated that sitting time was negatively correlated with physical activity (r = −0.250, *p* < 0.001) and positively correlated with arithmetic GPA (r = 0.167, *p* = 0.014) and wGPA (r = 0.185, *p* = 0.003).

Nomophobia was positively correlated with insomnia severity (r = 0.172, *p* = 0.011) and with MSWBI (r = 0.268, *p* < 0.001), while insomnia severity was positively correlated with MSWBI (r = 0.387, *p* < 0.001). Emotion-focused and avoidant coping strategies were significantly positively correlated with nomophobia, insomnia severity, and poorer well-being (all *p* < 0.05, Table 3). Furthermore, the emotion-focused coping strategy was positively correlated with both the problem-focused coping strategy (r = 0.563, *p* < 0.001) and the avoidant coping strategy (r = 0.296, *p* < 0.001).

Finally, academic achievement (wGPA) was negatively correlated with insomnia severity (r = −0.172, *p* = 0.011) and with avoidant coping strategies (r = −0.167, *p* = 0.014; Table 3).

### 3.4. Hierarchical Regression Analysis

In the first step of the hierarchical regression analysis (Table 4), demographic variables (age, gender, body mass index, and year of study) were entered into the model. This model explained 3.5% of the variance in wGPA (R^2^ = 0.035). None of the predictors reached statistical significance. In the second step, lifestyle variables (average daily sitting time and total physical activity) were added to the model. The inclusion of these variables increased the explained variance to 6.0% (R^2^ = 0.060). However, neither physical activity nor sitting time was a significant predictor at this stage. In the third step, digital and sleep-related variables (insomnia and nomophobia) were included. This resulted in a small increase in explained variance to 7.4% (R^2^ = 0.074), but neither variable significantly predicted wGPA. Finally, psychological variables (MSWBI and coping strategies) were added in the fourth step. The final model explained 9.0% of the variance in wGPA (R^2^ = 0.090). None of the predictors were statistically significant in the final model, although average daily sitting time approached statistical significance (*p* = 0.050, Table 5). Regression coefficients with corresponding 95% confidence intervals are presented in Table 5. Assessment of multicollinearity indicated no evidence of problematic collinearity among the predictor variables (VIF range: 1.14–3.64; tolerance range: 0.275–0.876).

## 4. Discussion

This study reveals a high prevalence of digital dependence and psychological vulnerability among medical students, with the majority demonstrating clinically relevant levels of nomophobia that were associated with insomnia severity and impaired well-being. Insomnia severity showed the strongest bivariate association with poorer well-being, thereby suggesting that insomnia symptoms may represent one potential link between technology overuse and psychological distress. Moreover, emotion-focused and avoidant coping strategies were associated with nomophobia, insomnia severity, and diminished well-being, delineating a coherent maladaptive coping profile. Furthermore, both insomnia severity and reliance on avoidant coping were negatively correlated with academic performance, suggesting modest bivariate associations with self-reported academic performance. The results of the regression analysis indicate that the examined demographic, lifestyle, digital, and psychological variables explained only a small proportion of the variance in students’ wGPAs. This relatively modest, explained variance highlights the complexity of academic achievement, which is influenced by numerous factors beyond those included in the present study. Variables such as study habits, actual study time, class attendance, intrinsic motivation, cognitive ability, emotional intelligence, learning strategies, social support, socioeconomic circumstances, and educational environment have all been associated with academic performance and may account for a substantial proportion of the unexplained variance. Therefore, the present findings should be interpreted as reflecting only one component of the multifactorial determinants of academic achievement.

The high prevalence of moderate to severe nomophobia in this cohort is consistent with recent epidemiological findings (Al-Mamun et al., 2025) and reflects the increasing centrality of smartphones in academic, social, and emotional domains (Bhattacharya et al., 2019). The positive association between nomophobia and insomnia severity may be interpreted through several complementary mechanisms. First, from a behavioral conditioning perspective, smartphone use may reinforce cognitive-emotional arousal through repeated social connectivity and information-access behaviors (Bhattacharya et al., 2019; Yildirim & Correia, 2015), particularly when these behaviors delay bedtime and disrupt sleep patterns (Oliveira et al., 2025). Second, circadian misalignment may arise from bedtime procrastination and blue light exposure, contributing to prolonged sleep latency and fragmented sleep (Oliveira et al., 2025). However, the association between nomophobia and insomnia symptoms should be interpreted cautiously, as the direction of this relationship remains unclear. While excessive smartphone use may negatively affect sleep through delayed sleep timing, increased cognitive arousal, and nighttime exposure to screen-based activities (Oliveira et al., 2025), the reverse pathway is also plausible. Students experiencing insomnia symptoms or psychological distress may rely more heavily on smartphones as a form of distraction, emotional regulation, or social interaction. Therefore, longitudinal studies are needed to clarify the temporal relationship between nomophobia and sleep disturbances. In addition, problematic smartphone use has been associated with poorer academic outcomes (Paterna et al., 2024), suggesting that digital over-engagement may operate as both a stress amplifier and a coping substitute. Within the transactional model of stress and coping (Lazarus & Folkman, 1984), nomophobia may reflect maladaptive appraisal processes in which perceived disconnection from the device triggers anxiety responses (Bhattacharya et al., 2019; Yildirim & Correia, 2015). The positive correlations observed between nomophobia and emotion-focused and avoidant coping further support this observation (Lazarus & Folkman, 1984; Eisenberg et al., 2012; Carver, 1997). Rather than addressing stressors directly (problem-focused coping), students with higher nomophobia may rely on emotion modulation or disengagement strategies, which are less effective in high-demand academic contexts (Eisenberg et al., 2012; Kassaw & Demareva, 2025).

Previous studies have demonstrated that sleep quality, duration, and regularity are robust predictors of academic performance in university students (Okano et al., 2019) and that sleep disturbances contribute substantially to academic underachievement (Hartmann & Prichard, 2018). Insufficient or fragmented sleep impairs executive functioning, working memory consolidation, attentional stability, and emotional regulation (Okano et al., 2019; Hartmann & Prichard, 2018). These domains are particularly critical in medical education, where sustained cognitive load and rapid knowledge integration are required (Hötting & Röder, 2013; Clemente-Suárez et al., 2023). Chronic sleep restriction may attenuate neurobiological adaptive processes, thereby diminishing cognitive performance. Moreover, sleep disturbance exacerbates affective dysregulation, which is highly prevalent among medical students (Rotenstein et al., 2016; Ishak et al., 2013), and may partly explain the strong association between insomnia severity and poorer MSWBI scores found in this study. Thus, insomnia may function as an important linking factor between behavioral dysregulation (e.g., excessive smartphone use), maladaptive coping, psychological distress, and academic outcomes. It should also be noted that the present study assessed only insomnia symptoms using the Insomnia Severity Index (ISI) and did not capture the broader, multidimensional nature of sleep health. Contemporary sleep research conceptualizes sleep health as a complex construct encompassing sleep duration, regularity, timing, efficiency, daytime alertness, and circadian alignment, as well as behavioral aspects such as social jetlag and compensatory weekend sleep. Therefore, our findings should be interpreted specifically in the context of insomnia symptoms rather than overall sleep health. Future studies should incorporate broader subjective and objective assessments of sleep, including longitudinal sleep monitoring, to better characterize the complex relationships among smartphone use, psychological well-being, coping strategies, and academic performance.

The significant positive associations of emotion-focused and avoidant coping with nomophobia, insomnia, and poorer well-being align with the foundational stress-appraisal-coping framework (Lazarus & Folkman, 1984). In high-demand environments such as medical school, coping strategies modulate the impact of stressors on psychological and functional outcomes. Avoidant coping has been shown to exacerbate functional impairment in clinical populations (Eisenberg et al., 2012) and may similarly undermine academic efficiency by delaying task engagement, increasing rumination, and triggering physiological stress. The negative correlations observed between wGPA and both emotion-focused and avoidant coping are consistent with psychosocial models identifying adaptive self-regulation as a key predictor of academic achievement (Kassaw & Demareva, 2025). Interestingly, emotion-focused coping was positively correlated with problem-focused coping, suggesting that coping repertoires are not mutually exclusive and multiple strategies can be deployed depending on situational appraisal (Lazarus & Folkman, 1984; Carver, 1997). However, reliance on maladaptive strategies in response to chronic academic stress may contribute to cumulative dysregulation, including sleep disruption and digital overuse (Eisenberg et al., 2012; Kassaw & Demareva, 2025).

The finding of approximately half of the participants with poor well-being is consistent with prior meta-analytic evidence of high psychological morbidity in medical students (Rotenstein et al., 2016) and documented burnout risk (Ishak et al., 2013). The association between insomnia severity and well-being highlights the potential relevance of insomnia symptoms to students’ psychological health, although causal or downstream effects cannot be inferred from the present design. Moreover, emerging research highlights the importance of psychological well-being and academic readiness in preventing attrition and optimizing performance in early medical training (Hefny et al., 2024).

Physical activity and sedentary behavior represent important components of students’ lifestyle patterns and were therefore considered in the present study. It has been shown that regular physical activity has been associated with better sleep quality, improved mood regulation, and enhanced cognitive functioning, whereas prolonged sedentary behavior, particularly when combined with extensive screen exposure, may contribute to poorer sleep habits and reduced psychological well-being (Firth et al., 2020; Bull et al., 2020). Furthermore, physical fitness has been positively associated with academic performance in student populations (Redondo-Flórez et al., 2022). Among university students, high academic demands and time spent studying may promote sedentary lifestyles, potentially reinforcing maladaptive coping patterns such as excessive smartphone use (Pengpid & Peltzer, 2019). However, in our sample, neither physical activity nor sedentary time showed significant associations with nomophobia, insomnia symptoms, well-being, or coping strategies. Although previous studies have suggested that physical activity and sedentary behavior may influence sleep, psychological well-being, and cognitive functioning, our findings indicate that these relationships may be more complex and potentially influenced by other individual, behavioral, and contextual factors. The absence of significant associations in the present study may also reflect the multifactorial nature of academic performance and psychological well-being among university students, where digital behavior, sleep, coping mechanisms, and physical activity may interact in more complex ways than can be captured by individual lifestyle components alone. Furthermore, the interpretation of sedentary behavior in this population deserves consideration. Although prolonged sedentary behavior has generally been associated with adverse health and cognitive outcomes, total sitting time measured by the IPAQ does not distinguish between academically engaged sedentary activities (e.g., attending lectures, studying, or completing coursework) and passive sedentary behaviors (e.g., recreational screen use). Consequently, greater sitting time in medical students may partly reflect greater academic engagement rather than exclusively unhealthy behavior. Therefore, the modest positive association observed between sitting time and academic performance should be interpreted with caution and should not be regarded as evidence of a beneficial effect of sedentary behavior itself. Future studies using longitudinal designs and objective assessments of physical activity and sedentary behavior, thereby distinguishing between active and passive sedentary activities, may help to better understand their potentially divergent relationships with sleep, psychological well-being, and academic achievement.

Due to the demanding nature of medical studies, characterized by a high academic workload and an intensive pace, medical students represent a relevant population for investigating how the combined influence of physical activity, insomnia, psychological resilience, stress-coping strategies, and smartphone use behaviors affects academic performance (Rotenstein et al., 2016; Hefny et al., 2024). They experience higher rates of depressive symptoms and suicidal ideation compared to the general population, as well as substantial levels of burnout (Rotenstein et al., 2016; Ishak et al., 2013). In parallel, sleep disturbances and insomnia are highly prevalent in this group and have been directly linked to poorer academic outcomes, further amplifying their vulnerability to stress-related impairment (Oliveira et al., 2025; Dąbrowska-Galas et al., 2021; Okano et al., 2019; Hartmann & Prichard, 2018). Moreover, problematic smartphone use and nomophobia are increasingly documented among university students and are negatively associated with academic achievement, adding a contemporary behavioral risk factor to an already high-stress educational context (Paterna et al., 2024; Bhattacharya et al., 2019; Al-Mamun et al., 2025).

In our hierarchical regression model, demographic variables (age, gender, BMI, and year of study) were included as potential confounding variables because previous studies have shown that they may influence academic achievement and psychological outcomes (Stallman, 2010; Richardson et al., 2012). Furthermore, lifestyle, digital, and psychological variables were then entered hierarchically according to our conceptual model.

This study possesses several notable strengths that enhance its scientific contribution. It captured a multidimensional profile of medical students by simultaneously assessing lifestyle behaviors (physical activity and sedentary time), technology-related behavioral patterns (nomophobia), sleep disturbances (insomnia severity), psychological well-being, coping strategies, and academic achievement. This integrative design enables a systems-level perspective rather than an isolated examination of single risk factors, thereby reflecting the complex biopsychosocial reality of medical education. The inclusion of students from all six years of medical training provides a comprehensive cross-sectional snapshot of academic exposure across different phases. The sample size was adequate for detecting small-to-moderate associations, and the instruments employed are internationally validated and widely used in comparable populations. The good to excellent internal consistency coefficients observed in this sample further strengthen measurement robustness. Importantly, the study bridges traditional lifestyle determinants of academic performance (such as physical activity and insomnia) with emerging digital-era constructs such as nomophobia, offering timely and contextually relevant insights.

However, several methodological considerations warrant careful interpretation of the findings. Foremost, the cross-sectional design precludes causal conclusions. Also, the study was conducted at a single medical school, which may limit generalizability. Further, exclusive reliance on self-reported data introduces the risk of recall bias, reporting inaccuracies, and social desirability effects. In particular, self-estimated physical activity and sedentary time are known to be prone to over- or underestimation, while perceived smartphone dependence may not directly correspond to objectively recorded usage patterns. Although all questionnaires were reviewed for completeness and consistency, no technical restrictions limiting responses to one Google account or IP address and no minimum completion time threshold were implemented. Therefore, the possibility of careless responding cannot be entirely excluded. Furthermore, the IPAQ assesses total sitting time and does not distinguish between academically engaged sitting (e.g., studying or attending lectures) and passive sedentary behaviors (e.g., recreational screen time), which should be considered when interpreting these findings. In addition, academic performance was assessed using self-reported GPA rather than official university records. Although this approach preserved participants’ anonymity, it may have introduced reporting or social desirability bias. Sleep assessment was limited to insomnia symptoms measured by the ISI. Other important dimensions of sleep health, such as sleep duration, sleep regularity, sleep timing, social jetlag, weekend catch-up sleep, and daytime sleepiness, were not evaluated, limiting a comprehensive assessment of sleep health. Although students from all six academic years were included, participation was voluntary and partly facilitated through in-class recruitment due to initial low response rates. This use of two recruitment strategies (online distribution and in-class recruitment) may have introduced participation or non-response bias. Potential differences between participants recruited online and those recruited through in-class invitations were not assessed and therefore cannot be excluded. Students experiencing higher levels of psychological distress, greater academic burden, or poorer academic performance may have differed in their willingness to participate, potentially leading to under- or overrepresentation of specific student subgroups. Therefore, the observed associations should be interpreted with appropriate caution. Additionally, potential confounding variables, such as socioeconomic status, baseline mental health diagnoses, caffeine or substance use, and personality traits, were not controlled for, which may have influenced the observed associations. Since the third gender category included only three participants, it was excluded from the final regression analysis to avoid unstable parameter estimates. This exclusion may have introduced a small degree of bias, although its impact on the overall findings is likely minimal given the very small number of participants. Although the sensitivity analysis indicated adequate statistical power to detect small-to-moderate overall regression effects, the study may have had insufficient power to identify very small independent effects of individual predictors. Finally, although statistically significant, the observed correlations were small in magnitude, suggesting that academic performance is influenced by a broader constellation of factors beyond the variables assessed in this study. Furthermore, although several examined factors showed associations with academic performance in bivariate analyses, the regression model did not identify any statistically significant independent predictors, and the explained variance was limited. Although conceptually related variables such as psychological well-being, insomnia symptoms, and coping strategies may share some common variance, multicollinearity diagnostics did not indicate problematic collinearity. Therefore, the lack of statistically significant independent predictors is more likely to reflect the modest explanatory power of the model and the multifactorial determinants of academic performance than statistical collinearity among the examined variables. Therefore, these findings should be interpreted as preliminary associations rather than evidence of factors that determine or drive academic achievement.

Future research should employ longitudinal designs, larger and more diverse samples, and incorporate objective or more comprehensive assessments, including actigraphy, sleep diaries, objectively recorded smartphone use, and verified academic records. The inclusion of additional cognitive, motivational, socioeconomic, educational, and environmental determinants would enable the development of more comprehensive models of academic performance. Such approaches would help clarify the temporal direction and potential causal relationships among digital behavior, insomnia symptoms, psychological well-being, coping strategies, and academic performance. Future studies should also examine the individual dimensions of nomophobia and coping strategies to identify specific behavioral profiles that may serve as targets for more personalized preventive interventions. In addition, objective monitoring of smartphone use and sleep parameters may help overcome limitations associated with self-reported measures and provide a more accurate understanding of these complex interactions. Finally, advanced analytical approaches, such as mediation analysis or structural equation modeling, may further elucidate the potential indirect pathways linking digital behavior, insomnia symptoms, psychological well-being, coping strategies, and academic performance.

## 5. Conclusions

In conclusion, this study identified significant associations among digital dependence, insomnia symptoms, maladaptive coping, psychological well-being, and academic performance in medical students. These findings suggest that academic performance is likely influenced by a broad range of cognitive, psychological, behavioral, social, and educational factors, only some of which were examined in the present study. However, given the cross-sectional design, the observed associations should not be interpreted as evidence of causal relationships. The observed associations of technology-related behaviors, insomnia symptoms, and coping styles highlight the potential value of integrated prevention strategies rather than isolated interventions. Early identification of maladaptive coping patterns and impaired psychological well-being may be valuable, as these factors may persist into later professional life. Our findings may help inform the development of preventive strategies, such as sleep hygiene education, resilience and coping skills training, promotion of physical activity, and responsible digital behavior as part of institutional support. The effectiveness of such interventions in improving academic performance and student well-being should be evaluated in future longitudinal and interventional studies.

## Figures and Tables

**Table 1:** Sociodemographic and academic characteristics of the sample.

Variable	Mean ± SD	N (%)
Age (years)	22.03 ± 2.48	
Gender		
Male		57 (26.39)
Female		156 (72.21)
Year of study		
1		49 (22.68)
2		33 (15.28)
3		36 (16.67)
4		30 (13.88)
5		22 (10.19)
6		46 (21.30)
Number of years of studying	4.26 ± 2.15	
GPA	4.16 ± 0.43	
wGPA	3.85 ± 0.55	
BMI (kg/m^2^)	22.59 ± 3.55	
Presence of chronic disease		31 (14.35)
Chronic medication use		27 (12.50)

GPA: Grade point average (arithmetic mean); wGPA: Weighted grade point average; BMI: Body mass index. Data are shown as means ± SD or N (%).

**Table 2:** Psychological and behavioral measures.

Variable	Mean ± SD	IQR
Total physical activity (MET-min/week)	3604.20 ± 3804.97	1063.88–4615.13
Daily sitting time (hours/day)	5.98 ± 2.41	4.5–7.5
Nomophobia total score	71.35 ± 25.43	56–87
ISI	7.32 ± 5.18	3–11
MSWBI	3.27 ± 1.89	2–5
Coping strategies		
Problem-focused	1.93 ± 0.56	1.63–2.38
Emotion-focused	1.68 ± 0.47	1.33–2.0
Avoidance	0.77 ± 0.46	0.38–1.0

IQR: Interquartile range; ISI: Insomnia severity index; MSWBI: Medical Student Well-Being Index.

**Table 3:** Correlation coefficients between psychological and behavioral measures and academic achievement.

Variable	Sitting Time	Nomophobia	ISI	MSWBI	Problem- Focused	Emotion- Focused	Avoidance	GPA	wGPA
Physical activity	−0.250 *	−0.074	−0.076	−0.033	0.061	−0.000	0.042	−0.095	−0.054
Sitting time		0.115	−0.007	0.130	0.029	0.098	0.103	0.167 *	0.185 *
Nomophobia			0.172 *	0.268 *	0.046	0.227 *	0.237 *	0.014	−0.028
ISI				0.387 *	−0.065	0.155 *	0.341 *	−0.128	−0.172 *
MSWBI					0.052	0.315 *	0.343 *	−0.052	−0.074
Coping strategies									
Problem-focused						0.563 *	−0.039	0.001	0.017
Emotion-focused							0.296 *	−0.118	−0.123
Avoidance								−0.157 *	−0.167 *
GPA									0.826 *

ISI: Insomnia severity index; MSWBI: Medical Student Well-Being Index; GPA: Grade point average (arithmetic mean); wGPA: Weighted grade point average. * *p* < 0.05.

**Table 4:** Hierarchical regression models predicting cumulative wGPA.

Model	Predictors Entered	R	R^2^
Model 1	Age, Gender, BMI, Year of study	0.187	0.035
Model 2	Model 1 + Sitting time, Physical activity	0.246	0.060
Model 3	Model 2 + ISI, Nomophobia	0.271	0.074
Model 4	Model 3 + MSWBI, Problem-focused, Emotion-focused, and Avoidance coping	0.300	0.090

BMI: Body mass index; ISI: Insomnia severity index; MSWBI: Medical Student Well-Being Index.

**Table 5:** Final hierarchical regression model predicting cumulative wGPA.

					95% Confidence Intervals	
Predictor	B	SE	t	*p*	Lower	Upper
Intercept	4.492	0.717	6.265	<0.001	3.071	5.912
Age	−0.039	0.036	−1.082	0.282	−0.110	0.032
Gender (male and female) *	0.065	0.119	0.546	0.586	−0.171	0.301
BMI	−0.011	0.017	−0.631	0.529	−0.044	0.023
Year of study	0.067	0.049	1.350	0.180	−0.031	0.165
Sitting time (h/day)	0.041	0.021	1.977	0.050	−0.0001	0.081
Physical activity	<0.001	<0.001	−0.220	0.826	−0.00003	0.00002
ISI	−0.009	0.010	−0.897	0.372	−0.029	0.011
Nomophobia	−0.001	0.002	−0.608	0.544	−0.005	0.003
MSWBI	0.019	0.027	0.685	0.495	−0.036	0.073
Problem-focused coping	0.133	0.115	1.155	0.250	−0.095	0.361
Emotion-focused coping	−0.146	0.135	−1.084	0.281	−0.414	0.121
Avoidance coping	−0.013	0.118	−0.114	0.910	−0.247	0.220

BMI: Body mass index; ISI: Insomnia severity index; MSWBI: Medical Student Well-Being Index. * Gender was coded as 1 = male and 2 = female and entered into the regression model as a binary categorical variable. The three participants who reported another gender identity were excluded from the regression analysis because their small number did not allow reliable estimation of regression coefficients.

## Data Availability

The data are contained within the article and the recordings and raw datasets supporting the conclusions of this study will be made available by the corresponding author on request.

## Abbreviations

The following abbreviations are used in this manuscript:

GPA	Grade Point Average
wGPA	Weighted Grade Point Average
BMI	Body Mass Index
ISI	Insomnia Severity Index
MSWBI	Medical Student Well-Being Index
MET	Metabolic Equivalent of Task
IQR	Interquartile Range
SD	Standard Deviation
SE	Standard Error
R^2^	Coefficient of Determination
